# The perilous state of seagrass in the British Isles

**DOI:** 10.1098/rsos.150596

**Published:** 2016-01-13

**Authors:** Benjamin L. Jones, Richard K. F. Unsworth

**Affiliations:** 1 Sustainable Places Research Institute, Cardiff University, Cardiff CF10 3BA, UK; 2 Project Seagrass, Sustainable Places Research Institute, Cardiff University, Cardiff CF10 3BA, UK; 3 Seagrass Ecosystem Research Group, College of Science, Swansea University, Wallace Building, Swansea SA2 8PP, UK

**Keywords:** eutrophication, indicators, seagrasses, submerged aquatic vegetation, water quality, Zostera

## Abstract

Seagrass ecosystems face widespread threat from reduced water quality, coastal development and poor land use. In recent decades, their distribution has declined rapidly, and in the British Isles, this loss is thought to have been extensive. Given increasing knowledge of how these ecosystems support fisheries production, the understanding of their potential rapid loss, and the difficulty in restoring them, it is vital we develop an understanding of the risks they are under, so that management actions can be developed accordingly. Developing an understanding of their environmental status and condition is therefore critical to their long-term management. This study provided, to our knowledge, the first examination of the environmental health of seagrass meadows around the British Isles. This study used a bioindicator approach and involved collecting data on seagrass density and morphology alongside analysis of leaf biochemistry. Our study provides, to the best of our knowledge, the first strong quantitative evidence that seagrass meadows of the British Isles are mostly in poor condition in comparison with global averages, with tissue nitrogen levels 75% higher than global values. Such poor status places their long-term resilience in doubt. Elemental nutrient concentrations and morphological change suggest conditions of excess nitrogen and probable low light, placing many of the meadows sampled in a perilous state, although others, situated away from human populations were perceived to be healthy. Although some sites were of a high environmental health, all sites were considered at risk from anthropogenic impacts, particularly poor water quality and boating-based disturbances. The findings of this study provide a warning of the need to take action, with respect to water quality and disturbance, to prevent the further loss and degradation of these systems across the British Isles.

## 1. Introduction

The eelgrass *Zostera marina* forms a highly productive habitat within temperate coastal ecosystems of the Northern Hemisphere [1]. In recent decades, meadows of *Z. marina* have declined rapidly [2,3] with anthropogenic activity thought to be accountable for the majority of the reported loses [3,4]. Globally, the loses of seagrass are occurring at an estimated rate of 7% yr^−1^ [4], and with an estimated 44% of the world’s population now living within 150 km of the ocean, these declines are only set to increase [5]. Learning how to protect these habitats is vital if we are to continue to reap the benefits of the ecosystem services they provide to humanity [6]. This requires creating a far greater understanding of the safe environmental boundaries of their existence in order to develop thresholds of safety for their management.

Increased nutrient input is one of the main forms of anthropogenic loading into the marine environment [7–9], and evidence suggests that this presents the biggest threat to seagrass meadows [4,10]. This over-enrichment causes shifts in primary production away from seagrass, towards faster-growing, nutrient-limited macroalgae, epiphytic microalgae and phytoplankton [11]. Numerous studies have reported how excessive growth of epiphytic algae on seagrass is a contributing factor to seagrass degradation through smothering and decreasing their ability to absorb light [12,13]. Increased input of nutrients enhances this excessive growth of epiphytes [14], reducing the resilience of seagrass and placing their viability at risk [10].

Owing to their susceptibility to changes in environmental conditions such as light and nutrients, seagrasses can be used as indicators of ecosystem quality and health [15]. Within the European Union (EU) Water Framework Directive (WFD), seagrasses are increasingly used in assessing the ecological status of coastal areas, where ecological status is classified by comparing characteristics, mainly shoot density, percentage cover or depth limits, to a reference level which reflects that of perceived low anthropogenic influence [16,17].

In the North Atlantic, seagrass meadows have recently been highlighted for their value as a nursery habitat for juvenile fishes of commercial value such as plaice and Atlantic cod [18–20], and their biodiversity value is recognized by their inclusion in Marine Habitat Action Plans, and as a focal part of the UK Biodiversity Action Plan [21].

In the British Isles (including the UK), seagrass meadows have historically been damaged and degraded [22,23], but detailed long-term data are spatially limited. We know that multiple anthropogenic drivers of change are present within these systems [22], and recent reviews [24] have categorized *Zostera* as being nationally scarce. Collation of sporadic datasets suggests populations are in severe decline (loss estimated at between 25% and 49% in the past 25 years) [25].

Available data on UK seagrass are also of limited scope and have limited capacity to reveal the environmental health of the meadow (e.g. mostly data on shoot density or total area). Although intertidal seagrasses in the UK have recently been included in government programmes, these EU WFD-focused assessments do not assess environmental conditions, neither via measurements of seagrass nutrient content or via physical-chemistry of the water column, and therefore provide us very little information pertinent to their long-term management. Importantly, there is little way of using the data to act as an early warning of future loss. Only the data collected within the Isles of Scilly monitoring programme presents information pertinent to understanding the status of the system as it is a long-term study that provides detailed data of shoot morphology, density, disease and epiphytic data [26].

Despite growing recognition for their ecosystem service value and an appreciation for their loss, there exists little detailed information about the environmental status of seagrass meadows throughout the British Isles. This is particularly the case with respect to nutrient enrichment which is highlighted as one of the key threats to these systems [22], and light availability which is a key driver of productivity [27]. Given that there is strong evidence that the water quality is poor in many areas of the British Isles known to contain seagrass [28], new data are therefore required that can attempt to determine the environmental health status of the seagrasses in the British Isles. This is particularly important, as we currently have no means of knowing whether seagrass meadows may already be very close to their environmental thresholds.

In many parts of the world, scientists and environmental managers are conducting seagrass monitoring studies that include the use of bioindicators to determine the environmental health of the seagrass system [29–31]. This approach uses the fact that seagrasses respond negatively to nutrient enrichment physiologically, morphologically and biochemically [27]. In fact, by examining seagrass bioindicators, there exists better scope to understand environmental condition as seagrass biochemical measurements are time-integrated rather than based on commonly used point-sampling methods for water quality assessments. By integrating such measurements with detailed quantification of their morphometrics and epiphytic algae, greater information about the environmental status of these sensitive yet important seagrass meadows can be collected.

The aim of this study was to provide the first rapid assessment of the ecological and environmental status of seagrass meadows (*Z. marina*) over a wide spatial scale throughout the British Isles using a series of established ecological and biochemical indicators. This study provides us with, to the best of our knowledge, the first use of biochemical indicators in seagrass meadows of the British Isles.

## 2. Material and methods

### 2.1 Study sites

**Figure 1.**
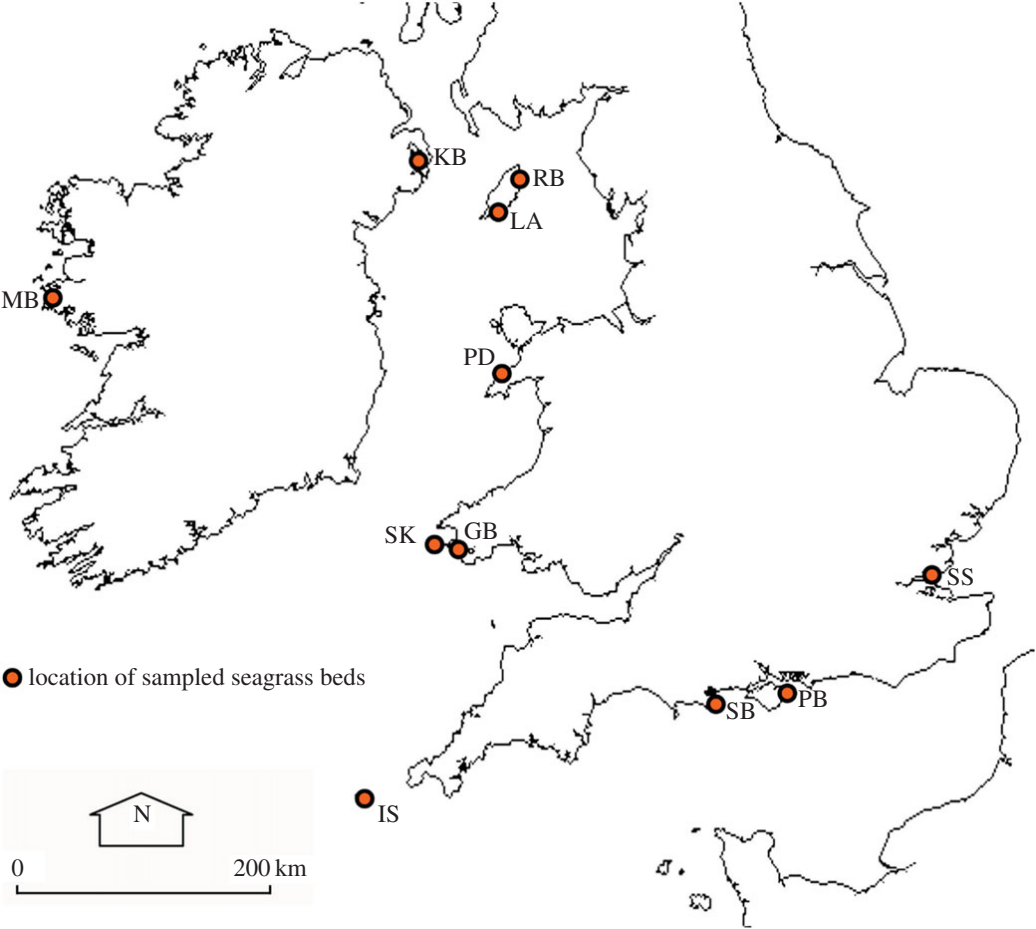
Seagrass meadows were sampled at 11 locations spread throughout the British Isles.

Seagrass meadows at 11 locations around the British Isles were assessed during May–August 2013 (table 1 and figure 1). Habitat data were collected in the field along with samples for further analysis in the laboratory. At each site, qualitative descriptive information was collected about the perceived presence of anthropogenic impacts in order to place the data in context. All seagrass sites were within the range of 0–3 m depth.

**Table 1.** Seagrass sampling sites around the British Isles, date of collection and initial assessment on perceived presence of anthropogenic impacts.

site	coordinates	date	industry	tourism	agriculture	catchment	population	anchoring	permanent mooring
Gelliswick Bay, Wales (GB)	51°42′26′′ N 5°3′42′′ W	26 May 2013	X	X	X	X	X	X	X
Southend-on-Sea, England (SS)	51°32′21′′ N 0°39′04′′ E	17 July 2013	X	X	X	X	X	X	X
Priory Bay, Isle of Wight (PB)	50°42′23′′ N 1°5′45′′ W	11 June 2013		X	X	X	X		
Ramsey Bay, Isle of Man (RB)	54°18′38′′ N 4°21′18′′ W	26 August 2013		X	X	X	X	X	
Studland Bay, England (SB)	50°38′34′′ N 1°56′28′′ W	17 July 2013		X	X	X	X	X	X
Kircubin Bay, Strangford Lough,	54°29′02′′ N 5°32′20′′ W	23 August 2013			X	X	X	X	
Northern Ireland (KB)									
Mannin Bay, Ireland (MB)	53°26′45′′ N 10°4′45′′ W	21 August 2013		X	X	X		X	
Langness, Isle of Man (LA)	54°4′27′′ N 4°36′48′′ W	18 June 2013		X	X			X	X
Porthdinllaen, Wales (PD)	51°44′30′′ N 5°16′79′′ W	27 June 2013		X	X			X	X
Isles of Scilly (IS)									
Little Arthur, Eastern Isles	49°56′59′′ N 6°15′59′′ W	28 July 2013		X				X	X
Higher Town Bay, St Martin’s	49°57′17′′ N 6°16′41′′ W	29 July 2013		X				X	X
Grimsby Harbour, Tresco	49°57′41′′ N 6°19′53′′ W	30 July 2013		X				X	X
Broad Ledges, Tresco	49°56′29′′ N 6°19′41′′ W	31 July 2013		X				X	X
Skomer MCZ, Wales (SK)	51°44′30′′ N 5°16′79′′ W	2 May 2013		X

### 2.2 Plant collection and morphological measurements

At all sampling sites, three haphazardly placed 0.25 m^2^ seagrass plots containing *Z. marina* were sampled. Shoot density counts and percentage cover estimates were taken on site and recorded. All seagrass material within the quadrats was then collected for subsequent laboratory analysis. Large macroalgae and *Zostera noltii* were not collected where it was present. Morphological measurements (leaf width and length, number of leaves per shoot) were taken in the laboratory.

Collected biomass was removed from sample bags and rinsed thoroughly in fresh water to remove salt water, sediment and detritus. Sheath length and width were taken from the longest intact leaf of each shoot. Length was measured using a rule to the nearest 1.0 mm from the meristem to the top of the leaf and width was measured with callipers to the nearest 0.05 mm. Number of leaves per shoot was also recorded for all shoots collected. All epiphytes, where present, were carefully scraped from both sides of the leaf using a microscope slide.

Cleaned leaf sections were dried at 60°C for 24 h and ground until homogeneous before dry leaf mass was recorded using an Ohaus balance (max 100 g;*d*=0.1 mg; Switzerland). Ground leaf tissue and shoot density were used to calculate individual shoot biomass for comparison purposes. Similarly, the scraped epiphytes were also dried at 60°C until they were of constant weight before their dry mass was recorded. Epiphyte mass was combined with shoot density to calculate epiphyte biomass per shoot.

### 2.3 Leaf carbon, nitrogen and phosphorus content

Ground seagrass leaf tissue was analysed for carbon (C), nitrogen (N) and phosphorus (P) [29]. Samples were analysed for per cent nitrogen and per cent carbon by weight using a continuous flow isotope ratio mass spectrometer (ANCA SL 20-20, Europa Scientific, Crewe, UK), and analysis of total P was undertaken by inductively coupled plasma atomic emission spectrometry (Liberty AX Sequential ICP-AES, Varian Inc., CA, USA). Dry matter determinations were carried out on each sample, so values could be reported as per cent dry weight, after which molar C : N, C : P and N : P values were calculated [29,31]. Our use of C : N in this study is owing to its use as a robust, early warning indicator of light reduction [27]. Similarly, C : P has been identified as an indicator of environmental P limitation [30–33] and N : P as an indicator of the balance in abundance of environmental N and P (the seagrass Redfield ratio defines N : P values of 25–30 as balanced abundance of both N and P compared with light availability) [32,34–36].

### 2.4 Data analysis

All morphological values, where described, are reported as mean±s.d. One-way ANOVA was conducted using SigmaPlot v. 12.3. Prior to analysis, data were tested for normality to meet the assumptions of parametric tests, in the event of failure to adhere to normality and homogeneity of variance, one-way ANOVA on ranks was conducted as an alternative.

Each variable measured different aspects of seagrass status, and to identify which of these presented most variability in the data a principal component analysis (PCA) was used [37–39]. Principal components (PCs) with eigenvalues greater than 1.0 were considered, and variable coefficients of less than or equal to 0.3 and greater than 0.3 in each component were selected as dominant.

## 3. Results

### 3.1 Seagrass morphometrics

**Figure 2.**
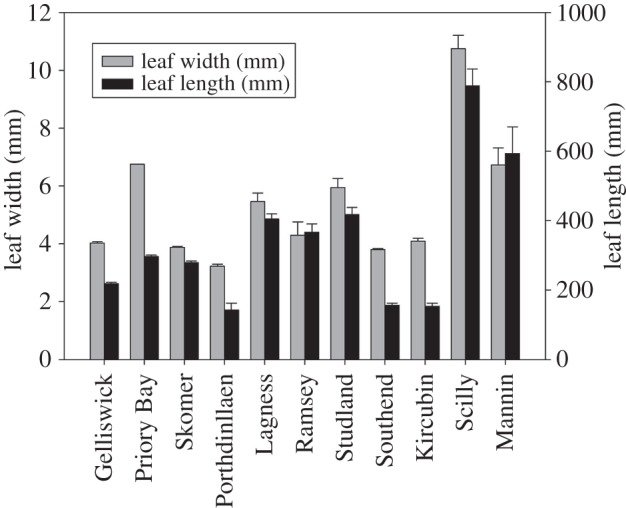
Leaf length and leaf width of seagrass at 11 locations spread throughout the British Isles.

Leaf length and width differed significantly between sites (figure 2 and table 2), with leaf length (*p*<0.001, *H*_10_=32.2) ranging from 142 to 788 mm (mean 347±201) and leaf width (*p*<0.001, *H*_10_=32.5) ranging from 3 to 11 mm (mean 5±2). Longest leaves were observed in the Isles of Scilly and Mannin Bay, and smallest in Porthdinllaen and Southend-on-Sea. Similarly, widest leaves were observed in the Isles of Scilly and Mannin Bay, with the narrowest being observed in Porthdinllaen. In addition, shoot characteristics were highly variable between sites. Shoot biomass also significantly (*p*<0.005, *H*_10_=27.9) differed with respect to site, ranging from 0.1 to 2.6 g (mean 0.3±0.8) with highest values in the Isles of Scilly and lowest values in Porthdinllaen, Skomer MCZ and Kircubin Bay. Shoot density was again significantly (*p*<0.005, *H*_10_=29.1) different between sites and ranged from 4.0 to 101.7 per 0.25 m^2^ (mean 48.3±32.4), with highest values in Priory Bay and Porthdinllaen, and lowest values in the Isles of Scilly and Ramsey Bay. Seagrass cover ranged from 16.7% to 91.3% (mean 48.1±21.4), with highest values in the Isles of Scilly and lowest values in Gelliswick Bay and Kircubin Bay. The number of leaves per shoot ranged from 3.5 to 6.0 (mean 4.6±0.9), with highest values in Priory Bay and lowest values in Mannin Bay and Porthdinllaen.

**Table 2.** Measured parameters of seagrass sampled at 11 locations spread throughout the British Isles.

site	%N	%P	shoot density (per/0.25 m^2^)	shoot biomass (g)	seagrass cover (%)	leaf length (mm)	leaf width (mm)	epiphytes (g per shoot)	leaves per shoot
Gelliswick Bay (GB)	4.37±0.36	0.27±0.06	44.0±10.4	0.09±0.04	16.7±2.9	218±4	4.0±0.0	0.06±0.02	4.0±0.0
Southend-on-Sea (SS)	5.00±0.18	0.33±0.01	31.7±3.5	0.08±0.01	50.0±0.0	156±7	3.8±0.0	0.00±0.00	3.7±0.1
Priory Bay (PB)	3.80±0.04	0.21±0.00	101.7±25.3	0.09±0.01	48.3±15.2	296±4	6.7±0.0	0.00±0.00	6.0±0.1
Ramsey Bay (RB)	3.18±0.21	0.19±0.03	18.7±1.2	0.09±0.02	55.0±5.0	367±24	4.3±0.5	0.00±0.00	4.9±0.4
Studland Bay (SB)	2.90±0.19	0.14±0.01	36.0±9.8	0.09±0.03	33.3±2.9	417±20	5.9±0.3	0.00±0.00	5.1±0.3
Kircubin Bay (KB)	3.35±0.21	0.18±0.03	85.0±38.0	0.06±0.01	20.0±8.7	153±9	4.1±0.1	0.00±0.00	4.3±0.2
Mannin Bay (MB)	2.27±0.22	0.14±0.03	42.0±14.7	0.19±0.12	65.0±26.5	594±77	6.7±0.6	0.01±0.01	3.5±0.3
Langness (LA)	3.43±0.51	0.17±0.02	25.0±3.0	0.14±0.01	61.7±2.9	405±14	5.5±0.3	0.01±0.00	5.6±0.1
Porthdinllaen (PD)	3.51±0.13	0.25±0.02	97.3±39.3	0.03±0.01	35.0±8.7	142±20	3.2±0.1	0.04±0.04	3.6±0.5
Isles of Scilly (IS)	2.63±0.29	0.13±0.01	4.0±1.4	2.61±1.40	91.3±2.5	788±49	10.7±0.5	0.00±0.01	5.4±0.3
Skomer MCZ (SK)	5.30±0.32	0.36±0.01	45.7±10.0	0.05±0.02	53.3±7.6	279±5	3.9±0.0	0.00±0.00	4.0±0.2
average	3.58±0.95	0.21±0.07	48.3±32.4	0.3±0.8	48.1±21.4	346±201	5.5±2.3	0.01±0.02	4.6±0.9

### 3.2 Nutrient concentrations and ratios

Significant differences in leaf tissue %N (*p*<0.001, *F*_10,32_=37.7) and %P (*p*<0.001, *F*_10,32_=29.8) were observed between locations. Per cent N ranged from 2.05 to 5.54 (mean 3.58±0.95), with highest values in Skomer MCZ and Southend-on-Sea and lowest values in the Isles of Scilly and Mannin Bay. These findings are surprising given that shoot density was highest at Porthdinllaen and Priory Bay, two sites of high %N. Pearson’s correlation found no significant relationship between %N and shoot density (electronic supplementary material, appendix 1). The %N values exhibited at sites in this study were mostly very high relative to other global values (table 3). Gelliswick Bay, Priory Bay, Skomer MCZ, Porthdinllaen, Langness, Ramsey Bay, Southend-on-Sea and Kircubin Bay all had values above 3% and only Studland Bay, the Isles of Scilly and Mannin Bay exhibited values below 3%. No sites exhibited %N values lower than the global average of 2.04%.

**Table 3.** *Zostera marina* leaf carbon, nitrogen and phosphorus content from literature sources.

location	%C	%N	%P	C : N	N : P	C : P	reference
North Carolina, USA	32.84	1.84	0.22	20.81	18.49	384.94	[40]
North Carolina, USA	33.67	1.53	0.25	25.66	13.53	347.31	[41]
Virginia, USA	37.50	2.20	0.22	19.88	22.11	439.57	[42]
Denmark	38.40	1.92		23.32			[43]
California, USA	38.40	2.37	0.34	18.90	15.41	291.25	[29]
South Korea	35.28	2.73		15.09			[44]
Denmark	30.78	1.94		19.44			[45]
Oregon, USA	34.00	2.70	0.40	14.69	14.93	219.20	[46]
Oregon, USA	35.00	1.30	0.20	31.40	14.37	451.29	[46]
Oregon, USA	29.00	1.40	0.10	24.16	30.96	747.85	[46]
various	36.00^a^	2.50^b^	0.39^c^	16.79	14.18	238.04	[32]
global average	34.62	2.04	0.27	20.92	18.00	389.93	
study averages	47.73	3.58	0.21	16.54	38.9	654.53	

^a^Forty-six measurements.

^b^Forty-five measurements.

^c^Thirty-six measurements.

Per cent P ranged from 0.11 to 0.38 (mean 0.21 ± 0.07), where highest values were recorded at Skomer MCZ and Southend-on-Sea, and lowest values in the Isles of Scilly and Studland Bay. Priory Bay, Porthdinllaen, Langness, Ramsey Bay, Studland Bay, Kircubin Bay, the Isles of Scilly and Mannin Bay all exhibited P values lower than the global average of 0.27% and only Gelliswick Bay, Skomer MCZ and Southend-on-Sea exhibited higher than average values.

**Figure 3.**
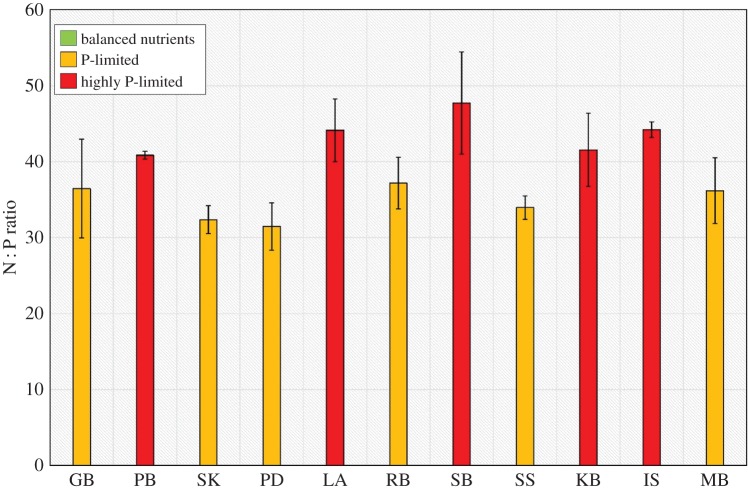
Leaf N : P ratio variability within seagrass at 11 locations spread throughout the British Isles (balanced nutrients = 0–20; P-limited = 20–40; highly P-limited ≥40) (values for differentiation between states derived from [30,31,33]).

**Figure 4.**
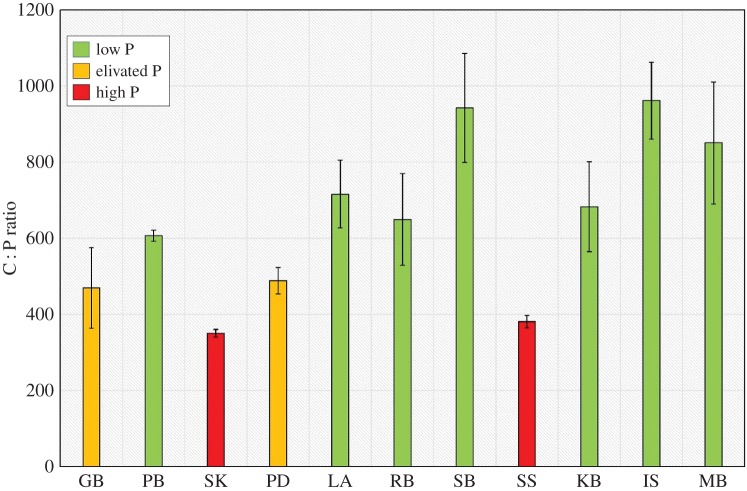
Leaf C : P ratio variability within seagrass at 11 locations spread throughout the British Isles (low P ≥600; elevated P = 400–600; high P ≤400) (values for differentiation between states derived from [30,31,33]).

Levels of the nutrient ratio N : P significantly differed with respect to site (*p*<0.001, *F*_10,32_=5.75). All sites were found to be highly imbalanced in terms of available nutrients with N : P values more than 30 (figure 3). Specifically, Priory Bay, Langness, Studland Bay, Kircubin Bay and the Isles of Scilly all had highly elevated N : P ratios (more than 40), suggesting high nutrient imbalance and a limitation in P. These sites, all with %P values lower than the global average also exhibited high C : P ratios (more than 500) indicating that the plants are growing in environments with a relatively low P pool (figure 4). C : P ratios also differed significantly with respect to site (*p*<0.001, *F*_10,32_=15.0). In comparison, Skomer MCZ, Southend-on-Sea and Gelliswick Bay, all with high P levels, elevated N : P and C : P ratios of less than 500, indicate that the plants are growing in a nutrient-rich environment with a relatively large P pool.

**Figure 5.**
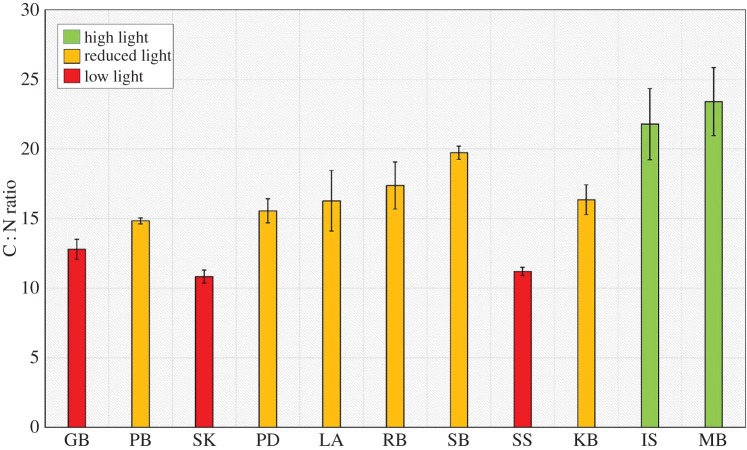
Leaf C : N ratio variability within seagrass at 11 locations spread throughout the British Isles (high light ≥20; reduced light = 14–20; low light ≤14) (values for differentiation between states derived from [30,31,33]).

The ratio of C : N can provide a potential indication of light limitation in seagrass [47], where levels below 20 suggest reduced light availability and levels below 15 being of potential limitation (figure 5) [27,33]. Levels of C : N differed significantly with respect to site (*p*<0.001, *F*_10,32_=22.8). On this basis, seagrass at Skomer MCZ, Southend-on-Sea and Gelliswick Bay all suffered from potential light limitation. Only the seagrass at the Isles of Scilly, Mannin Bay and to a lesser extent Studland Bay had levels suggesting sufficient light availability.

### 3.3 Principal component analysis

In order to determine which characteristics contributed most to the variability within and between sites, a PCA was used. In the PCA of the measured seagrass characteristics, there were three PCAs that had eigenvalues greater than 1 and together accounted for 78.4% of the variance (table 4). The first principal component (PC1), and the component with the most importance, had an eigenvalue of 5.37 and contributed to 53.7% of the variance. This component was dominated by molar C : N, molar C : P, percentage cover, shoot biomass, leaf width and leaf length. This axis is suggested to be loosely representative of growth and productivity, and thus overall health in relation to available nutrients and light. This is a useful way of ranking sites based on their leaf characteristics where an increase in PC1 represents an increase in overall leaf size attributed to an increase in light availability (high C : N) and a decrease in P load (high C : P).

**Table 4.** PCA of seagrass sampled at 11 locations spread throughout the British Isles. (Coefficients shown in italics represent dominant variables in each PC.)

	principal component		
	PC1	PC2	PC3
summary values			
eigenvalues	5.37	1.39	1.08
per cent variation	53.7	13.9	10.8
cumulative per cent variation	53.7	67.6	78.4
seagrass characteristics			
C : N	*0*.*344*	−0.024	*0*.*369*
C : P	*0*.*373*	−0.223	*0*.*375*
N : P	0.275	−*0*.*495*	0.253
per cent cover	*0*.*313*	0.271	−*0*.*453*
shoot biomass	*0*.*323*	*0*.*314*	−0.049
shoot density	−0.211	−*0*.*422*	−0.017
leaf length (mm)	*0*.*406*	0.209	0.032
leaf width (mm)	*0*.*403*	0.086	−0.034
epiphytes (g per shoot)	−0.175	*0*.*363*	*0*.*592*
leaves per shoot	0.25	−*0*.*414*	−*0*.*313*

Molar N : P, shoot biomass, shoot density, epiphytes per shoot and leaves per shoot were the dominant variables for the second principal component (PC2), which had a much smaller eigenvalue of 1.39 and contributed to an additional 13.9% of the variance. We identified PC2 as being representative of relative nutrient balance owing to the high weighting of molar N : P which, when low is indicative of balanced nutrients. The third and final principal component (PC3), which was responsible for a smaller 10.8% of the variance and had an eigenvalue of 1.08, was dominated by molar C : N, molar C : P, per cent cover, epiphytes per shoot and leaves per shoot. We can identify that lower light availability and increased nutrient load are indicative of lower seagrass coverage, and higher epiphyte biomass, but contribute less to the variability over each location.

**Figure 6.**
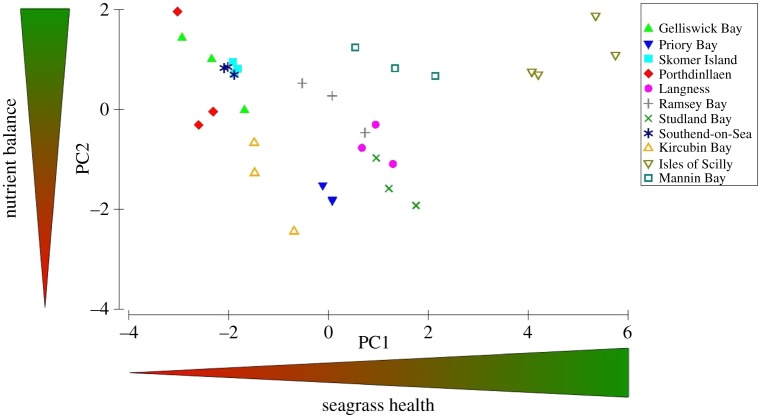
Principal component analysis (PCA) of seagrass characteristics from 11 locations spread throughout the British Isles.

Figure 6 illustrates the variability of seagrasses at sites in relation to PC1 and PC2. The spread of data along the PC1 axis (representative of growth as a measure of seagrass health) suggests that seagrass in the Isles of Scilly are in an ecologically healthy state compared with seagrasses located elsewhere in the British Isles, specifically that found in Southend-on-Sea, Gelliswick Bay, Skomer MCZ and Porthdinllaen.

### 3.4 Anecdotal observations

At all sites, available information and personal observations of the authors provided evidence of the presence of risk factors to seagrass. The majority of the sites assessed were found to have issues that have the potential to result in elevated nutrients conditions and disturbance. These risks may ultimately result in reduced water quality and light availability. Six of the sites were observed to have boat anchoring and permanent moorings present or in close proximity to the seagrass.

## 4. Discussion

Seagrass meadows are productive coastal habitats of high ecosystem service value that are threatened globally [2,4,48]. One of the main threats is that of declining water quality [4,11]. This study provides, to our knowledge, the first quantitative evidence of how such declining water quality is negatively impacting the health status of seagrass meadows throughout the British Isles. Although the data only provide a rapid and limited snap shot of the environmental status of these meadows, it is sufficient to clearly indicate that many seagrass meadows in the British Isles are under anthropogenic stress and probably in a poor state of health, many of which are in sites of apparent conservation protection (e.g. within special areas of conservation). The study also highlights the presence of some healthy seagrass meadows, but these are far from large human populations (Isles of Scilly and Mannin Bay, Ireland). Long-term ecological data from the Isles of Scilly seagrass confirm the relatively healthy status of these systems [26].

The tissue nutrient data for seagrasses of the British Isles collected in this study indicate that these habitats are highly over-enriched, with %N levels over 75% higher than the global average for *Z. marina*. Our British Isles sites had %N levels consistently higher than the global average with the exception of Isles of Scilly and Mannin Bay in the west of Ireland.

In comparison with %N levels, seagrasses were found to be on average low in %P with levels way below the global average, suggesting P-limitation and a largely unbalanced nutrient regime. P limitation may be exacerbated by the abundance of calcium carbonate sediments at some of these sites [49], particularly those in the Isles of Scilly, Studland Bay and Mannin Bay. Only seagrasses at Skomer MCZ and Southend-on-Sea had %P levels above the global average.

Specific sites of concern with high nitrogen values were those in Gelliswick Bay (Milford Haven), Priory Bay (Isle of Wight), Southend-on-Sea and Skomer MCZ. Although the first three of these are within or close to water bodies of known eutrophication problems (Milford Haven Waterway, Thames Estuary and Solent/Isle of Wight), the site at Skomer MCZ was initially thought to be removed from sources of high nutrient input. The capacity for leaves to absorb nutrients declines with leaf age, where highest N values have been observed in April (younger leaves), after which they rapidly decline to their lowest values in July [50]. Skomer MCZ and Gelliswick Bay were sampled at the start and end of May respectively, thus high leaf N levels may partly be owing to early sampling; however, subsequent samples taken at different times of the year have revealed similar levels of N (R Unsworth 2014, unpublished data) suggesting this is not the case. Additionally, we hypothesize that the high nutrient concentrations recorded at Skomer MCZ may be the result of the high populations of seals [51], birds [52] and potentially a small level of untreated effluent from nearby buildings present at the site. These issues may be exacerbated by potential low mixing of the water within the bay. As is increasingly being realized globally, seagrasses that contain a healthy and balanced associated fauna may actually be buffered from the impacts of elevated nutrients through the process of grazing [10,53]. The enhanced protection afforded to this site by MCZ designation may contribute towards buffering of these nutrient concerns.

The use of the C : N ratio as an indicator of light availability [27,54] also suggests that seagrass meadows of the British Isles are under anthropogenic stress, with values 25% lower than the global average and at levels that other studies have suggested indicate light limitation [27,30,54]. Particular concern exists for seagrass at Skomer MCZ, Priory Bay, Gelliswick Bay, Porthdinllaen and Southend-on-Sea which all have C : N ratios equal to or lower than 15 which is a level potentially below which is indicative of light limitation [27,30,31]. Not surprisingly, seagrass at the Isles of Scilly and Mannin Bay all had C : N levels higher than 20, suggesting a high light environment suitable for productive seagrass meadows. Studland Bay was also found to have a suitable light environment.

Low-impact (high C : N; high C : P) sites, such as the Isles of Scilly and Mannin Bay, were characteristic of high biomass, larger leaves, high numbers of leaves per shoot, high percentage cover, generally low shoot density and lower epiphyte biomass, whereas high-impact (low C : N; low C : P) sites, such as Gelliswick Bay, Southend-on-Sea and Skomer MCZ, were characteristic of low biomass, smaller leaves, low percentage cover, generally high shoot density and high epiphyte biomass showing similarities to previous findings elsewhere, with the exception of shoot density [55–57].

Our use of multivariate analysis enabled a generalized assessment of the ‘health’ of these 11 seagrass sites in the British Isles. All three sites in Wales (Gelliswick Bay, Skomer MCZ and Porthdinllaen) as well as that at Southend-on-Sea performed the worst. Although the present assessment provides an important warning about the status of seagrass at those sites the limited data extent provided by this rapid assessment requires that these findings are interpreted with caution. The data in this study were collected over a summer period between May and August, such unbalanced sampling may have influenced the data to a small degree owing to seasonality. Future comparative studies between sites using the indicators described in this study need to be undertaken with seasonal comparison and/or using consistent sampling times and use consistent methodologies.

Present research also highlights the value of using seagrass tissue nutrients and morphometric measurements as indicators of environmental change in these important ecosystems, reflecting ambient nutrient levels better than point sampling alone. Daily, seasonal and annual variability in water quality is caused both by pulsed events (e.g. outfall discharges, storms) and mixing through the action of tides and currents [58]. This, in addition to the relatively rapid uptake of nutrients by algae, microbes and plants [58–60] means that early signs of enrichment are difficult to detect and that point measurements of nutrients (conventional water samples) do not always describe the levels of nutrients available to the biota and its impacts. Seagrasses absorb nutrients from the water column and sediments; hence, seagrass leaf tissue nutrients can give a time-integrated relative measure of the available environmental nutrients [11,61,62].

Although the use of seagrass as an indicator has been readily applied as such a tool elsewhere in Europe [17], current seagrass monitoring strategies in the UK, which are routinely carried out, lack context and do not give indication of environment status as a whole [16,17]. The development of an index of seagrass health based on the main PC from PCA revealed that there was extensive variability between sites in the UK driven by parameters not considered in existing monitoring programmes. Our analysis identified that molar C : N and C : P, along with shoot biomass, leaf length and leaf width contributed to over 50% of the variability over the spread of data. Molar C : N, which indicates light availability (and N availability) and C : P which is indicative of P availability are useful indicators of environment quality [32]. Thus, by combining these with the morphological characteristics of *Z. marina*, we can begin to form a picture of how changes in environment quality, owing to nutrient enrichment, affect *Z. marina* health.

The present dataset suggests that C : N, C : P, leaf length and width as well as percentage cover are best used to determine ecological status of seagrass meadows around the British Isles. Shoot density has been readily applied as a measurement for meadow health [63], and is reported to respond negatively to nutrient enrichment [64–66]. This study, however, in addition to on-site observations, has shown that this partly does not hold true for the British Isles. The seagrass at Porthdinllaen, which shows signs of overenrichment, has relatively high shoot density and forms a large meadow. However, leaf length and width were the lowest recorded and epiphyte coverage was high. When compared with the seagrass at the Isles of Scilly, which has the lowest shoot density, but highest leaf length and width and low epiphyte coverage, it is clear that multiple seagrass metrics are required to assess the environmental and ecological health of the meadow.

In addition to the quantitative assessment of their environmental health, our study also revealed extensive qualitative anecdotal evidence of the impacts placed on seagrass meadows around the British Isles. Seagrass meadows at all sites except for Priory Bay and Skomer MCZ were subject to either permanent moorings, extensive anchoring or both. The extensive nature of the anthropogenic activity suggested other impacts (trampling and bait digging) were also present.

This study provided, to our knowledge, the first rapid environmental assessment of seagrass meadows throughout the British Isles. In conclusion, we find strong evidence of the perilous state of the majority of seagrass meadows sampled in the British Isles. These meadows were mostly subjected to excess nutrients and turbid conditions and even those not subject to poor water quality were subjected to other anthropogenic risks (e.g. moorings and anchoring). With increasing sea surface temperatures owing to climatic change, it is imperative that seagrass has sufficient light availability to maintain a positive carbon balance owing to elevated respiration [67]. What we are observing here suggests many seagrass meadows are potentially already close to their light thresholds and therefore, the resilience of these systems to such climatic change is being placed in doubt [10].

This study also shows that the seagrass *Z. marina* can readily be used to provide a valid representation of ecosystem status, more so than that of just water sampling alone. It is suggested that C : N, C : P, leaf length and leaf width, as well as per cent cover and shoot biomass are used for future monitoring of seagrass in the British Isles to give a time-integrated representation of meadow status.

Given the increasing body of evidence of the high fisheries nursery value of seagrass meadows throughout Northern Europe and the consequences of this for food security, this study provides evidence for the increasing need to manage the environmental condition of seagrass systems in the British Isles. Reducing the impact of poor water quality and disturbance will enhance the resilience of these systems into the future.

## Supplementary Material

Appendix 1 - All seagrass data collected from eleven seagrass meadows throughout the British Isles

## Supplementary Material

Appendix 2

## Data accessibility

All data collected in the present study are available within the electronic supplementary material appendix 2.
