# Supplementary material for: The perilous state of seagrass in the British Isles
Source: R Soc Open Sci. 2016 Jan 13;3(1):150596. doi: 10.1098/rsos.150596 (PMC4736943; doi:10.1098/rsos.150596)

Appendix 1. Relationship between seagrass (*Zostera marina*) leaf tissue Nitrogen and shoot density across 11 locations around the British Isles.

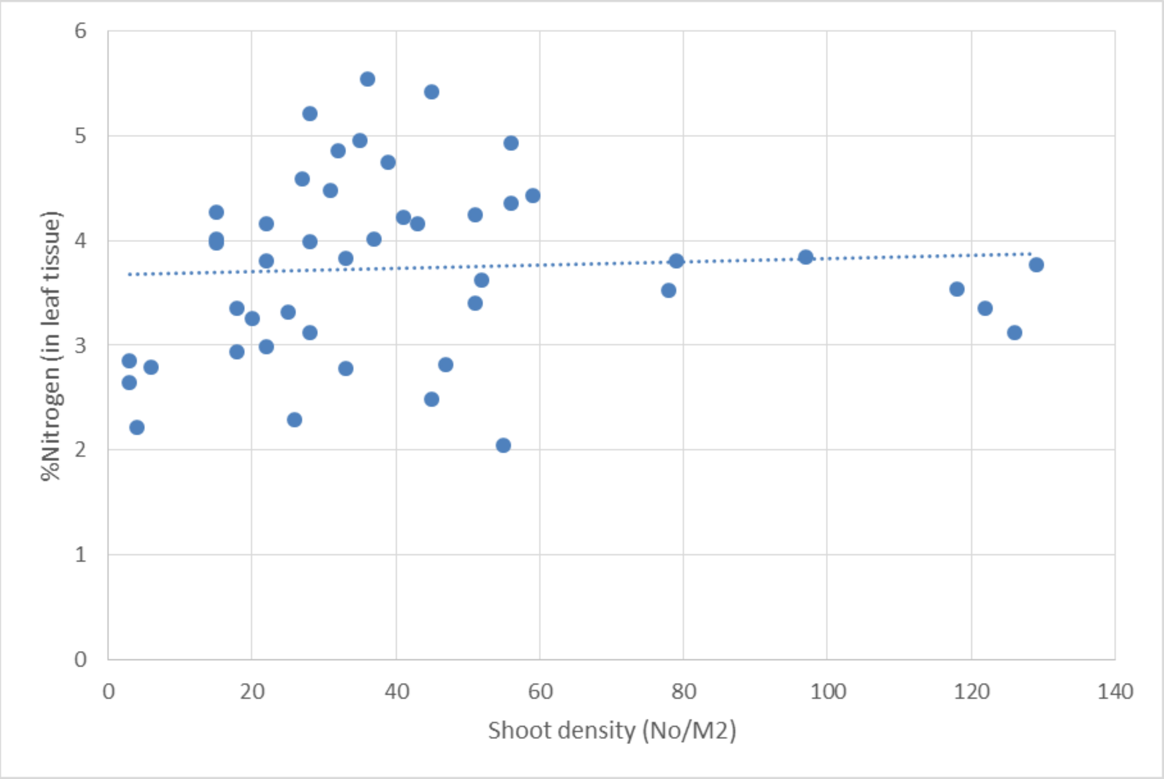

Supplement: Appendix 1 - All seagrass data collected from eleven seagrass meadows throughout the British Isles [file rsos150596supp1.pdf]
