# Supplementary material for: The perilous state of seagrass in the British Isles
Source: R Soc Open Sci. 2016 Jan 13;3(1):150596. doi: 10.1098/rsos.150596 (PMC4736943; doi:10.1098/rsos.150596)
Supplement: Appendix 2 [file rsos150596supp2.pdf]

Sample	P (% d/w)	N (%	C:N	C:P	N:P	% Cover	Shoot Biomas	Shoot Density	Leaf Length (mm	Leaf Width (m	piphytes (g/shoo	Leaves per shoot
G30	0.33	4.36	12.13	352.10	29.03	20	0.05	56	223.05	4.07	0.0600	3.98
G31	0.22	4.02	13.56	558.25	41.16	15	0.08	37	218.04	4.01	0.0343	4.00
G32	0.27	4.74	12.72	498.20	39.17	15	0.13	39	214.38	4.00	0.0836	3.97
PB01	0.21	3.77	14.98	607.60	40.57	65	0.10	129	300.36	6.75	0.0012	5.97
PB02	0.21	3.80	14.59	591.33	40.52	35	0.08	79	296.38	6.75	0.0004	5.89
PB03	0.21	3.84	14.96	619.98	41.44	45	0.08	97	292.41	6.74	0.0007	6.10
SK01	0.35	4.93	11.35	352.22	31.04	60	0.03	56	281.96	3.87	0.0048	4.15
SK02	0.36	5.54	10.42	359.15	34.47	45	0.07	36	280.93	3.84	0.0025	3.83
SK03	0.38	5.41	10.75	339.56	31.59	55	0.06	45	273.36	3.92	0.0038	3.93
PD01	0.26	3.36	16.56	474.67	28.67	45	0.03	122	165.90	3.30	0.0177	4.17
PD02	0.22	3.54	15.15	528.28	34.86	30	0.02	118	130.51	3.18	0.0201	3.29
PD03	0.26	3.62	14.97	462.35	30.89	30	0.04	52	130.80	3.18	0.0917	3.42
LA01	0.19	3.99	13.79	632.52	45.87	65	0.13	28	405.91	5.15	0.0046	5.61
LA02	0.17	2.99	17.87	704.36	39.42	60	0.15	22	418.61	5.74	0.0036	5.61
LA03	0.16	3.31	17.18	809.36	47.11	60	0.15	25	390.15	5.48	0.0068	5.70
RA01	0.16	2.94	19.24	788.13	40.96	55	0.08	18	380.19	4.20	0.0011	5.13
RA02	0.21	3.26	16.94	582.83	34.41	50	0.08	20	338.65	3.89	0.0055	4.47
RA03	0.20	3.35	15.97	577.03	36.13	60	0.11	18	381.65	4.81	0.0022	5.12
SB01	0.15	2.78	19.87	817.02	41.12	30	0.12	33	425.60	6.22	0.0021	5.44
SB02	0.13	2.82	19.21	911.35	47.45	35	0.05	47	432.12	6.03	0.0013	5.15
SB03	0.13	3.12	20.13	1098.54	54.56	35	0.10	28	394.22	5.60	0.0018	4.65
SS01	0.32	4.85	11.55	388.33	33.63	50	0.07	32	150.12	3.83	0.0028	3.58
SS02	0.34	4.95	11.09	361.96	32.63	50	0.08	35	153.97	3.80	0.0029	3.80
SS03	0.32	5.21	11.00	391.86	35.63	50	0.07	28	163.60	3.79	0.0025	3.68
KB01	0.15	3.12	17.54	817.58	46.62	30	0.07	126	163.62	4.20	0.0001	4.46
KB02	0.20	3.41	16.12	596.72	37.03	15	0.05	51	147.50	3.99	0.0002	4.29
KB03	0.19	3.53	15.45	633.94	41.03	15	0.06	78	148.15	4.08	0.0003	4.15
IOS01	0.14	2.65	21.49	923.66	43.08	90	4.44	3	788.53	10.95	0.0033	5.33
IOS02	0.14	2.79	20.44	923.26	45.17	90	1.42	6	743.36	10.69	0.0050	5.07
IOS03	0.14	2.86	19.75	888.99	44.97	95	1.60	3	764.62	10.13	0.0067	5.69
IOS04	0.11	2.21	25.46	1111.32	43.64	90	2.99	4	855.77	11.21	0.0025	5.54
MB01	0.12	2.05	26.00	962.74	37.01	85	0.13	55	622.00	7.04	0.0065	3.71
MB02	0.13	2.29	23.03	921.98	40.04	35	0.32	26	652.22	7.09	0.0188	3.11
MB03	0.17	2.49	21.16	666.87	31.51	75	0.11	45	506.80	6.04	0.0038	3.70
